# Flatline Thromboelastography During Massive Intraoperative Bleeding in a Patient With Multiple Myeloma: A Case Report

**DOI:** 10.7759/cureus.113486

**Published:** 2026-07-27

**Authors:** Nivedithaa, Soma Ganesh Raja Neethirajan, Akilandeswari M

**Affiliations:** 1 Anaesthesia, Sri Ramachandra Institute of Higher Education and Research, Chennai, IND; 2 Anesthesiology, Sri Ramachandra Institute of Higher Education and Research, Chennai, IND

**Keywords:** coagulopathy, flat line in thromboelastography, fresh frozen plasma, massive bleeding, multiple myeloma, point of care coagulation testing

## Abstract

Multiple myeloma is a malignant plasma cell disorder associated with complex coagulation abnormalities that may increase the perioperative bleeding risk. We report the case of a 38-year-old man with multiple myeloma, chronic kidney disease on maintenance haemodialysis, thrombocytopenia, and recent clopidogrel use, who underwent posterior spinal decompression and instrumented fusion from C7 to T6 for an epidural abscess. Intraoperatively, he developed massive hemorrhage with an estimated blood loss of 5,000 mL, accompanied by profound hemodynamic instability requiring vasopressor support. Thromboelastography (TEG) showed a flatline tracing, indicating near-complete failure of clot formation. Prompt recognition of the coagulopathy and aggressive goal-directed transfusion with 5 units of packed red blood cells, 10 units of fresh frozen plasma, and 10 units of platelets resulted in successful hemostatic resuscitation and stabilization. The coagulopathy was likely multifactorial, due to a combination of hemostatic dysfunction associated with multiple myeloma, uremic platelet dysfunction, thrombocytopenia, recent antiplatelet therapy, and ongoing surgical bleeding. This case highlights the importance of viscoelastic testing in identifying severe coagulopathy in patients with plasma cell malignancies and guiding perioperative transfusion management.

## Introduction

Multiple myeloma is a plasma cell malignancy characterized by the clonal proliferation of plasma cells and excessive production of monoclonal immunoglobulins. Although thromboembolic complications are well-recognized in patients with multiple myeloma, bleeding manifestations may also occur as a consequence of complex and multifactorial hemostatic abnormalities involving paraproteins, platelets, coagulation factors, fibrinogen function, and the vascular endothelium [1-3]. Bleeding may arise from thrombocytopenia, qualitative platelet dysfunction, acquired von Willebrand syndrome, coagulation factor inhibition, paraprotein-associated dysfibrinogenemia, and uremic platelet dysfunction.

The bleeding tendency associated with multiple myeloma has been attributed to a variety of mechanisms, including thrombocytopenia, qualitative platelet dysfunction, acquired coagulation factor deficiencies, dysfibrinogenemia, and the presence of circulating heparin-like anticoagulants [1-3]. Importantly, these abnormalities may not be adequately detected by conventional coagulation tests, such as prothrombin time (PT), activated partial thromboplastin time (aPTT), and platelet count, potentially leading to underestimation of the true hemostatic derangement. Conventional coagulation tests may not fully capture the complexity of hemostatic abnormalities in patients with multiple myeloma. These investigations evaluate individual components of the coagulation system in isolation and provide only a limited snapshot of hemostasis. As a result, serious abnormalities, such as qualitative platelet dysfunction, impaired clot strength, defective fibrin polymerisation, and fibrinolytic disturbances, may remain unrecognized. In addition, paraprotein-related interference and uremic platelet dysfunction can significantly impair haemostasis despite relatively preserved conventional coagulation parameters.

Viscoelastic assays, such as thromboelastography (TEG), provide a comprehensive real-time assessment of clot initiation, propagation, clot strength, and fibrinolysis. By offering a global evaluation of coagulation dynamics, TEG facilitates the early identification of coagulopathy and enables targeted, goal-directed transfusion therapy in patients experiencing major haemorrhage [4,5].

This report aims to describe the perioperative management of profound intraoperative coagulopathy in a patient with multiple myeloma undergoing major spinal surgery and to highlight the role of TEG-guided transfusion in the management of massive haemorrhage.

## Case presentation

**Figure 1:**
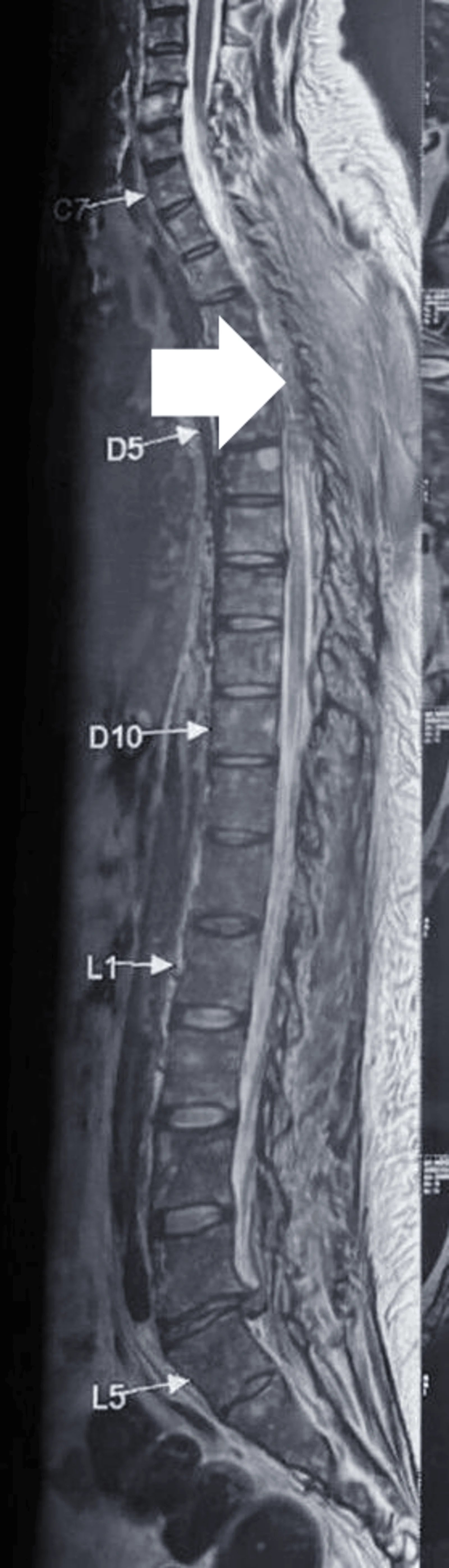
Magnetic resonance imaging of the spine showing an epidural abscess extending from C7 to D6. Arrow indicating epidural abscess.

A 38-year-old hypertensive male with ischemic heart disease on clopidogrel 75 mg OD has had multiple myeloma for 1.5 months on cyclophosphamide, bortezomib, and dexamethasone (CYBORD regimen). He was on thrice-weekly dialysis for chronic kidney disease. His last dialysis was heparin-free dialysis done 16 hours prior to surgery. He presented with progressive numbness and weakness of both lower limbs along with urinary incontinence for five days. Magnetic resonance imaging (MRI) of the lumbosacral spine with whole-spine screening demonstrated signal-intense lesions involving all the vertebrae, femur, and pelvis. Posterior disc protrusion with dorsal disc migration was noted, causing indentation of the ventral thecal sac with narrowing of the neural foramina and impingement of the traversing nerve roots at the L3-L5 levels. Disc desiccation with reduced intervertebral disc height was observed at the C4-C7 levels. An epidural abscess extending from the C7 to T6 levels was identified. Figure 1 demonstrates the epidural abscess on the MRI.

Preoperative laboratory investigations demonstrated anaemia, leukopenia, thrombocytopenia, renal dysfunction, and mild coagulation abnormalities. The laboratory findings are summarized in Table 1.

**Table 1:** Preoperative laboratory values PT: prothrombin time; aPTT: activated partial thromboplastin time; SGPT: serum glutamate pyruvate transaminase; SGOT: serum glutamic oxaloacetic transaminase

PARAMETER	PATIENT VALUE	REFERENCE RANGE
Hemoglobin	9.1 g/dl	12-17 g/dl
Total leukocyte count	3490 cells/cu mm	4000-11000 cells/cu mm
Platelet	118 x 10⁹/L	150-450 x 10⁹/L
PT	15.3 seconds	Control: 12.2 seconds
aPTT	31.1 seconds	Control: 24.2 seconds
INR	1.33	0.9-1.4
Blood urea nitrogen	49 mg/dl	7-20 mg/dl
Serum creatinine	7.9 mg/dl	0.6-1.2 mg/dl
Sodium	134 mmol/L	135-45 mmol/L
Potassium	4.8 mmol/L	3.5-5.0 mmol/L
Chloride	104 mmol/L	98-106 mmol/L
Bicarbonate	15 mmol/L	22-28 mmol/L
Total bilirubin	0.73 mg/dl	0.3-1.2 mg/dl
Total protein	8.3 g/dl	6.5-8.5 g/dl
Albumin	2.5 g/dl	3-4.5 g/dl
SGPT	33 U/l	0-35 U/l
SGOT	18 U/l	0-45 U/l

**Figure 2:**
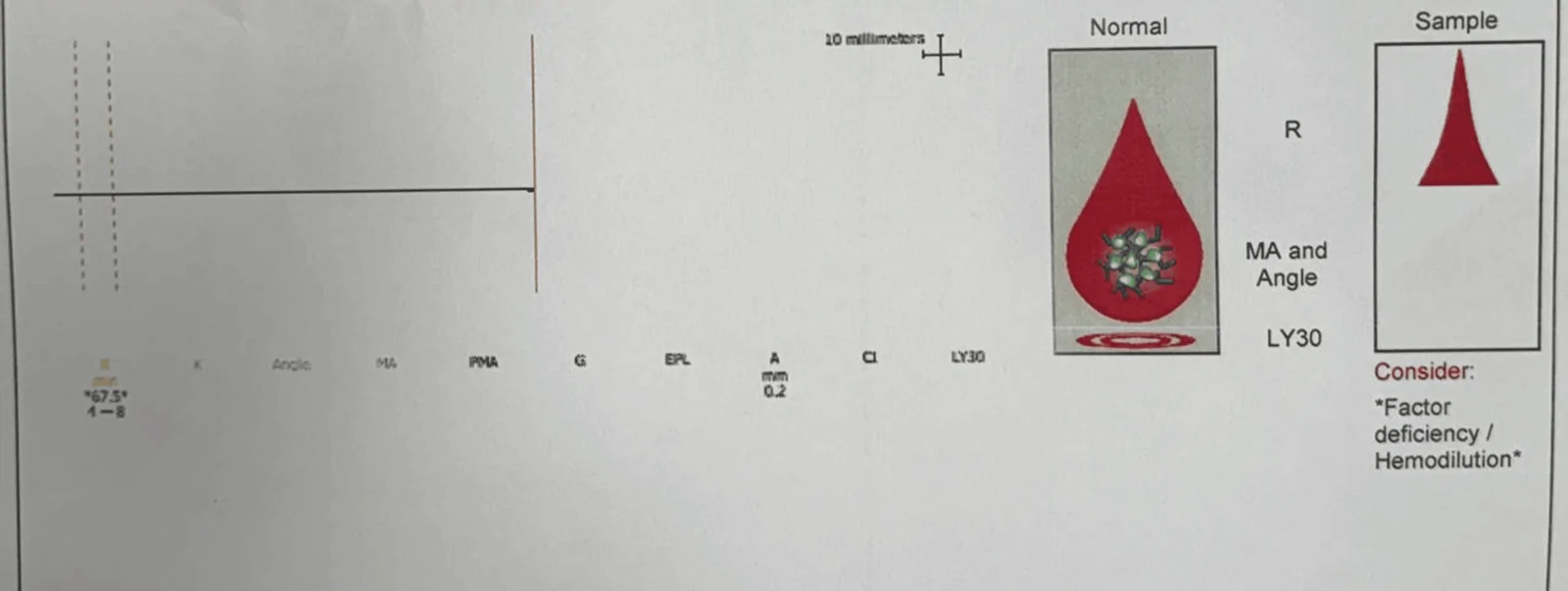
Intraoperative thromboelastography showing a flatline tracing with no measurable clot formation

After obtaining informed consent, he was scheduled for posterior spinal decompression and instrumented fusion from C7 to D6 under American Society of Anesthesiologists (ASA) physical status IV. Blood products were prepared in advance, including 3 units of packed red blood cells (PRBCs), 4 units of fresh frozen plasma (FFP), and 10 units of random donor platelets (RDPs). General anesthesia was induced with fentanyl (2 µg/kg), propofol (1 mg/kg), and atracurium (0.5 mg/kg). The trachea was intubated with an 8.0 mm flexo-metallic endotracheal tube. An arterial line was secured in the left radial artery. Prior to surgical incision, 1 g of tranexamic acid and four units of FFPs were administered. Baseline arterial blood gas analysis (ABG) revealed pH 7.35, PaCO₂ 35.3 mmHg, PaO₂ 87.2 mmHg, hemoglobin 8.42 g/dL, sodium 140.4 mmol/L, potassium 5.23 mmol/L, chloride 106.3 mmol/L, calcium 1.06 mmol/L, and lactate 0.77 mmol/L. After positioning the patient prone, surgery commenced. It was ensured that the abdomen was free. Within 30 minutes of incision, blood loss of approximately 800 mL was noted, following which four units of RDPs were transfused due to baseline thrombocytopenia and surgical bleeding; blood was sent for TEG. In the event of persistent hypotension, the patient was started on noradrenaline infusion. The initial viscoelastic assay demonstrated a flatline TEG, following which two units of RDPs, two units of FFP, and two units of PRBCs were transfused. Figure 2 demonstrates the flatline TEG. Table 2 demonstrates the parameters in the first TEG. Intraoperative TEG was performed using the TEG 5000 hemostasis analyzer with a kaolin-activated assay, in accordance with the manufacturer's recommendations and institutional protocol. Whole-blood samples were analysed within five minutes of collection to minimize preanalytical variability. After the initial flatline TEG, the test was repeated to exclude technical errors.

**Table 2:** Parameters in TEG 1

Parameter	Value	Unit	Normal Range
R time	Not measurable	minutes	4-8
Alpha angle	Not measurable	degrees	47-74
Maximum amplitude (MA)	Not measurable	mm	55-73

**Figure 3:**
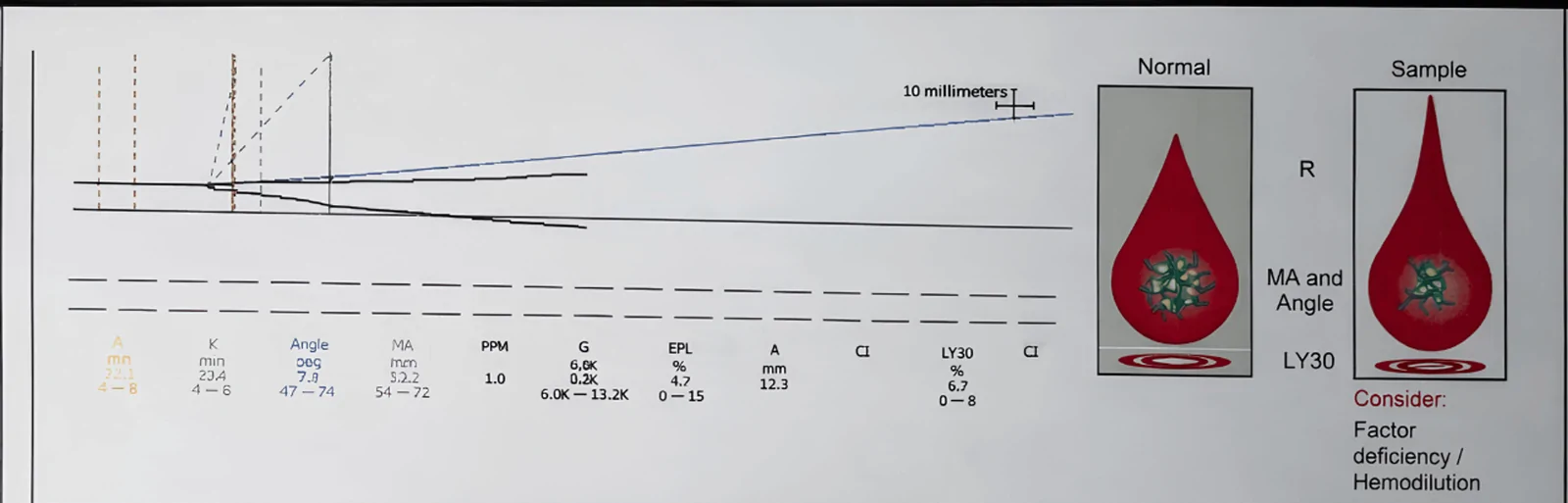
Thromboelastography showing an R time of 22.3 minutes

Activated clotting time was prolonged at 278 seconds. A repeat TEG revealed a flatline. Additional transfusion included four units of FFP, four units of RDPs, and three units of PRBCs. Repeat TEG performed after transfusion of 10 units of RDPs, 10 units of FFP, and 5 units of PRBCs revealed a prolonged R time of 22.3 minutes and a maximum amplitude of 10.6 mm. Serum fibrinogen levels were assessed, and they were found to be 567 mg/dL (normal range is 250-520 mg/dL). Although the assay method was not documented, the preserved fibrinogen concentration does not exclude qualitative fibrinogen dysfunction or impaired fibrin polymerisation. The procedure lasted four hours and was complicated by massive haemorrhage, with an estimated blood loss of 5,000 mL, representing approximately 78% of the patient's estimated blood volume (6.4 L). Hemostatic resuscitation included transfusion of 5 units of PRBCs (1,800 mL), 10 units of FFP (1,890 mL), and 10 units of RDPs (600 mL), in addition to 800 mL of Ringer's lactate. Intravenous calcium (2 g) was administered during resuscitation to counteract transfusion-related hypocalcaemia. Normothermia was maintained throughout the procedure using forced-air warming and warmed intravenous fluids. Total urine output was 75 mL. Cell salvage was not available at our institution; therefore, blood replacement relied entirely on allogeneic blood products. The final ABG showed pH 7.37, PaCO₂ 39.7 mmHg, PaO₂ 344.8 mmHg, sodium 143.8 mmol/L, potassium 5.09 mmol/L, chloride 103.8 mmol/L, bicarbonate 22.7 mmol/L, calcium 1.14 mmol/L, lactate 2.19 mmol/L, and glucose 175.5 mg/dL. Figure 3 demonstrates TEG done after the transfusion of all blood and blood products and shows an R time of 22.3 minutes; the parameters are tabulated in Table 3.

The profoundly abnormal TEG findings reflected impairment across multiple stages of hemostasis. The prolonged R time (22.3 minutes) suggested delayed clot initiation, consistent with coagulation factor deficiency or the presence of circulating anticoagulant activity. The markedly reduced alpha angle (7.7°) indicated impaired clot propagation, raising the possibility of defective fibrin polymerisation or functional fibrinogen abnormalities despite a preserved plasma fibrinogen concentration. The severely reduced maximum amplitude (10.6 mm) reflected markedly diminished clot strength, likely resulting from the combined effects of thrombocytopenia, qualitative platelet dysfunction related to uremia and multiple myeloma, paraprotein-mediated interference with clot formation, and ongoing massive haemorrhage. Rather than implicating a single mechanism, the TEG findings supported the presence of a profound, multifactorial coagulopathy.

**Table 3:** Parameters in TEG 2

Parameter	Value	Unit	Normal Range
R time	22.3	minutes	4-8
Alpha angle	7.7	degrees	47-74
Maximum amplitude (MA)	10.6	mm	55-73

**Figure 4:**
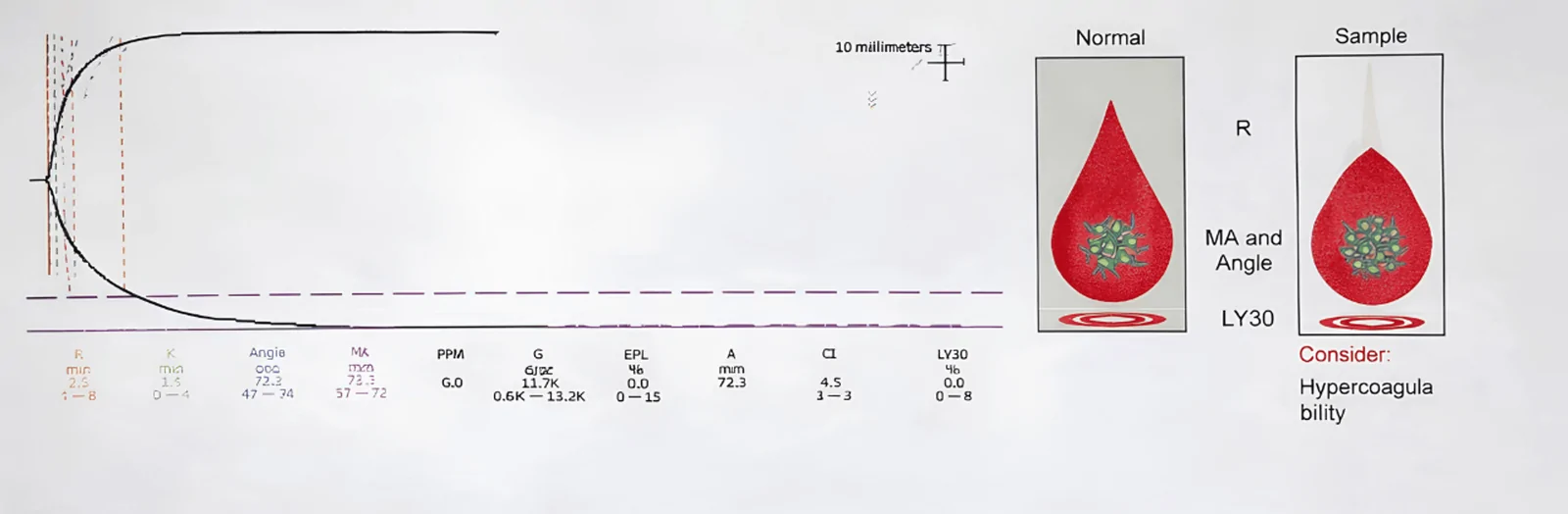
TEG done six hours post procedure

A repeat TEG performed six hours postoperatively demonstrated normalization of coagulation parameters with an R time of 2.5 minutes, an alpha angle of 72.2 degrees, and a maximum amplitude of 73.3 mm. Figure 4 demonstrates TEG done six hours post-procedure.

**Table 4:** Parameters in TEG 3

Parameter	Value	Unit	Normal Range
R time	2.5	Minutes	4-8
Alpha angle	72.2	Degrees	47-74
Maximum Amplitude (MA)	73.3	mm	55-73

Table 4 demonstrates parameters in the final TEG done six hours post-procedure. The normalization of TEG parameters six hours postoperatively likely reflected the combined effects of hemostatic resuscitation and restoration of physiological homeostasis rather than any single intervention. No additional blood products or dialysis was required during this period. By this time, definitive surgical hemostasis had been achieved, transfusion-related hypocalcemia had been addressed with intravenous calcium supplementation, and normothermia had been maintained using forced-air warming and warmed intravenous fluids. Collectively, these measures, together with cessation of ongoing blood loss and progressive haemodynamic stabilisation, likely contributed to the recovery of coagulation function observed on the postoperative TEG. The surgical procedure was completed successfully as planned. Following surgery, the patient was transferred intubated to the intensive care unit for elective postoperative mechanical ventilation and close hemodynamic monitoring. Serial clinical assessment and laboratory investigations demonstrated progressive hemodynamic stabilization and recovery of coagulation function, with normalization of TEG parameters six hours postoperatively. The patient was successfully weaned from ventilatory support and extubated once hemodynamically stable; no transfusion-related complications were observed, and the patient was neurologically appropriate. No new neurological deficits or major postoperative bleeding complications were observed. The subsequent ICU and ward stay were uneventful, and the patient was discharged in a stable condition with appropriate follow-up. Table 5 gives a consolidation of the TEG values of the three intraoperative TEGs.

**Table 5:** Consolidation of TEG parameters

PARAMETER	Flatline TEG	TEG 2	TEG after 6 hours	Unit	Normal Range
R time	Not measurable	22.3	2.5	minutes	4-8
Alpha angle	Not measurable	7.7	72.2	degrees	47-74
Maximum amplitude (MA)	Not measurable	10.6	73.3	mm	55-73

## Discussion

Overt bleeding is relatively uncommon in patients with multiple myeloma; however, complex pathophysiological interactions among paraproteins, coagulation factors, platelets, and the vascular endothelium can result in significant hemostatic disturbances. Paraproteins may impair platelet function through antibody-mediated coating of platelets and interfere with clotting factors I, II, V, VII, and VIII. In addition, antibodies directed against specific coagulation factors, endothelial dysfunction, and inhibition of fibrin polymerisation further compromise clot formation. Thrombocytopenia, either secondary to chemotherapy or due to bone marrow infiltration by malignant plasma cells, adds to the bleeding risk. Less frequently, circulating heparin-like anticoagulants may be present, typically manifesting as prolongation of both prothrombin time and activated partial thromboplastin time. Paraprotein-induced dysfibrinogenemia [3] resulting from structural and functional alterations of fibrinogen and acquired factor VIII deficiency may also contribute to a clinically significant bleeding diathesis [1,2].

In our patient, the coagulopathy was most likely multifactorial, reflecting the combined effects of multiple myeloma, chronic kidney disease with uremic platelet dysfunction, baseline thrombocytopenia, recent clopidogrel exposure, and ongoing massive haemorrhage. However, coagulation factor assays, platelet function testing, von Willebrand factor analysis, and heparinase-modified TEG were not performed.

Hemostatic abnormalities in multiple myeloma are therefore multifactorial and often unpredictable. TEG offers a rapid and comprehensive assessment of global hemostasis by evaluating clot initiation, propagation, strength, and fibrinolysis. Parameters such as R time and MA assist in identifying coagulation factor deficiencies and qualitative platelet dysfunction. In the present case, the intraoperative finding of a flatline TEG tracing, indicating absence of clot formation, reflected profound global coagulopathy and explained the massive haemorrhage encountered. This real-time information facilitated immediate, goal-directed transfusion of blood and blood components, ultimately restoring clot formation and achieving hemodynamic stability. The flatline TEG reflected severe coagulopathy, while its underlying mechanism remained multifactorial and incompletely defined. Several studies have demonstrated that TEG-guided resuscitation reduces overall blood product utilisation while improving targeted correction of coagulopathy and helping prevent ongoing bleeding [4,5].

Tranexamic acid was administered early as part of our hemostatic resuscitation strategy. Although the CRASH-2 trial established the benefit of early tranexamic acid administration in trauma-related hemorrhage [6], evidence specific to spine surgery has also demonstrated that tranexamic acid significantly reduces intraoperative blood loss and transfusion requirements without a consistent increase in thromboembolic complications when used appropriately [7,8]. Beyond its established role in trauma, tranexamic acid has emerged as an important component of patient blood management strategies in major surgery. Ongoing research, including the TATRA trial, continues to evaluate its effectiveness in reducing perioperative blood loss and transfusion requirements, underscoring the growing recognition of antifibrinolytic therapy as a valuable adjunct in the management of surgical hemorrhage [9].

Fibrinogen, the most abundant coagulation factor, is often the first to reach critically low levels during severe hemorrhage, and the degree of hypofibrinogenemia correlates closely with blood loss and adverse outcomes. Despite massive intraoperative hemorrhage, our patient had a preserved plasma fibrinogen concentration; consequently, fibrinogen concentrate was not administered. However, a normal fibrinogen concentration does not necessarily reflect normal fibrinogen function. The markedly reduced alpha angle on TEG suggested impaired fibrin polymerization or qualitative fibrinogen dysfunction, possibly related to paraprotein-mediated interference despite adequate circulating fibrinogen levels. As functional fibrinogen testing was not performed, the presence of a qualitative fibrinogen abnormality could not be confirmed, representing an important limitation of this report.

Activated clotting time (ACT) was prolonged in our patient despite the absence of heparin exposure. As a non-specific whole-blood assay, ACT can be prolonged by qualitative platelet dysfunction and coagulation factor deficiencies, both well-recognized features of multiple myeloma-associated coagulopathy. Moreover, protamine administration itself may prolong ACT and impair platelet aggregation, potentially worsening an existing coagulopathic state. Thus, empiric protamine administration based solely on a prolonged ACT in the absence of confirmed heparinisation may exacerbate bleeding rather than correct it. Hence, protamine was not administered despite a raised ACT. The patient underwent heparin-free hemodialysis before surgery, making residual dialysis-related heparin exposure unlikely. However, as heparinase-modified TEG was not performed, the possibility of an occult heparin effect could not be definitively excluded

Following induction, the patient received 1 g of tranexamic acid and four units of FFP before the surgical incision because of pre-existing coagulation abnormalities. Within 30 minutes of surgery, approximately 800 mL of blood loss had occurred, prompting TEG, transfusion of four units of RDP, and initiation of a noradrenaline infusion in response to haemodynamic instability. The initial TEG demonstrated a flatline tracing, following which an additional two units of RDP, two units of FFP, and two units of PRBC were administered, and ACT was measured. Repeat TEG remained flatline, while ACT was prolonged at 278 seconds. In view of the persistent severe coagulopathy and ongoing haemorrhage, a further four units of FFP, four units of RDP, and three units of PRBC were transfused. Subsequent TEG demonstrated recovery of clot formation, with a prolonged R time of 22.3 minutes, indicating partial restoration of coagulation. Serial TEG findings, interpreted alongside ongoing blood loss, hemodynamic status, and the surgical field, were used to guide successive transfusion decisions throughout the procedure.

Viscoelastic testing has emerged as an important tool for the assessment and management of perioperative coagulopathy. By providing a dynamic evaluation of the entire coagulation process, TEG overcomes several limitations of conventional coagulation tests and facilitates goal-directed transfusion therapy [4,5]. Evidence suggests that viscoelastic-guided transfusion protocols can optimize blood product administration, improve hemostatic correction, and reduce unnecessary transfusions during major surgery and massive hemorrhage [4,5]. Previous reports have described severe bleeding secondary to acquired dysfibrinogenemia and heparin-like anticoagulants in patients with multiple myeloma [3]. Desai et al. described a patient with multiple myeloma who developed severe bleeding secondary to a circulating heparin-like anticoagulant, highlighting the heterogeneous mechanisms responsible for coagulopathy in plasma cell dyscrasias [10]. Our case further illustrates how profound coagulopathy may manifest despite only mildly abnormal baseline coagulation studies.

**Figure 5:**
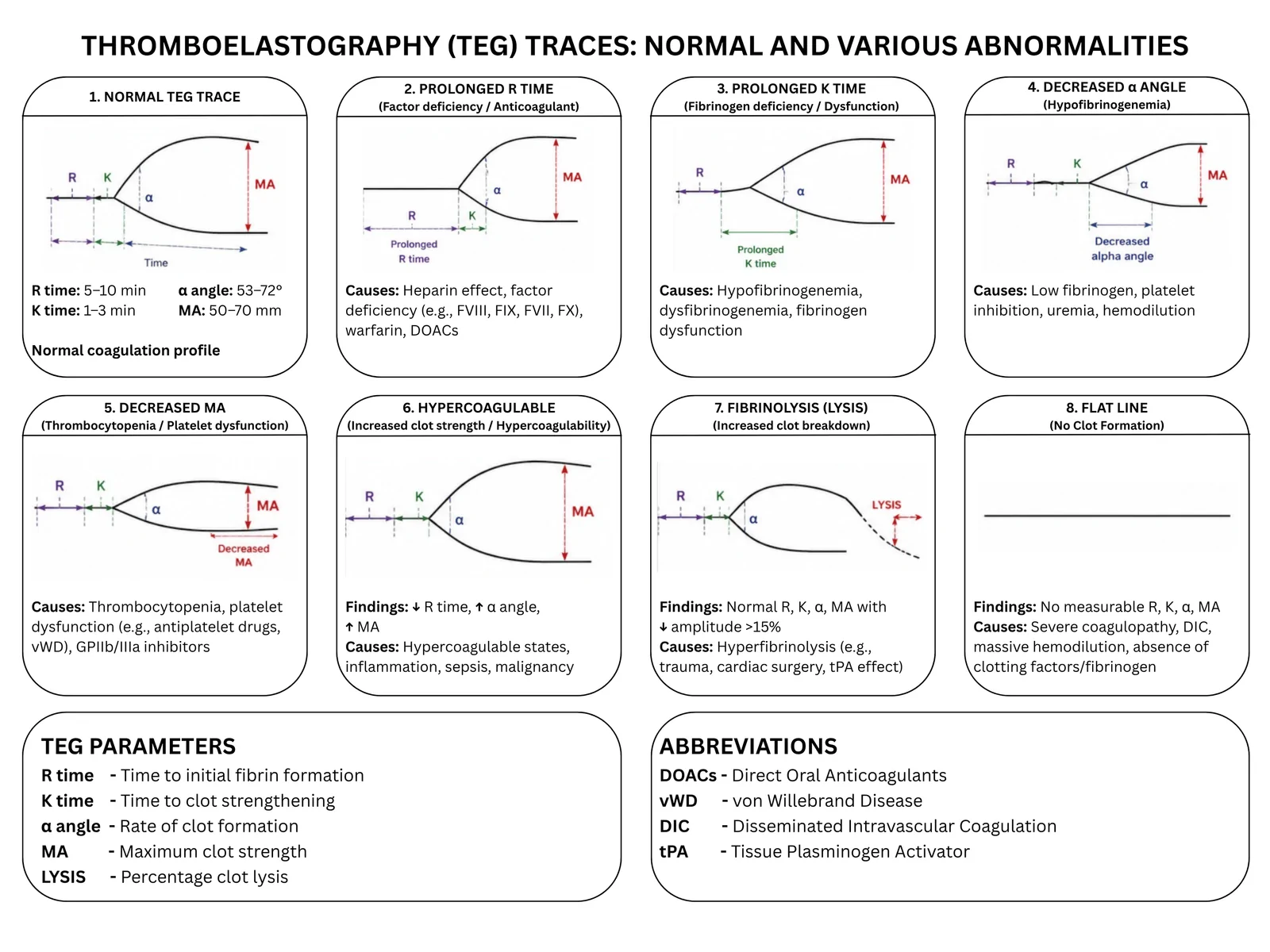
Schematic diagram of thromboelastography showing normal trace and various abnormalities Image credit: The authors; generated using Canva Pty Ltd., Sydney, Australia

This case report emphasizes the pivotal role of point-of-care coagulation testing in guiding transfusion during massive hemorrhage. In our patient, early intraoperative use of TEG enabled goal-directed transfusion, contributing to effective hemostatic resuscitation while avoiding unnecessary fluid and blood product administration, thereby reducing the risk of fluid overload. The initial and repeat TEG demonstrated a flatline tracing with non-measurable parameters; individual component-directed therapy based on isolated TEG abnormalities like prolonged R time or reduced MA was not possible. Instead, the flatline tracing was interpreted as evidence of profound global coagulopathy, prompting balanced replacement of coagulation factors, platelets, and red blood cells while serial TEG was used to assess recovery of clot formation. However, this case also highlights that preoperative TEG assessment might have identified underlying coagulation abnormalities and helped anticipate the severity of intraoperative hemorrhage. Figure 5 presents a schematic diagram of TEG depicting various abnormalities.

## Conclusions

This case highlights the value of thromboelastography (TEG) in the early recognition of severe perioperative coagulopathy and in guiding goal-directed haemostatic resuscitation during major spine surgery in a patient with multiple myeloma. The improvement in clot formation and hemodynamic stability likely resulted from targeted blood component therapy, definitive surgical hemostasis, calcium replacement, maintenance of normothermia, and restoration of physiological homeostasis. Although the individual contribution of each intervention cannot be determined, integrating viscoelastic testing with clinical assessment and conventional laboratory investigations facilitated informed transfusion decisions during massive hemorrhage. The case also underscores the importance of anticipating massive transfusion and incorporating perioperative monitoring with viscoelastic assays, activated clotting time (ACT), and serum fibrinogen measurements in patients with plasma cell malignancies. This multimodal approach may optimize hemostatic management and improve perioperative outcomes in this high-risk population.
